# Transformer-based operon prediction using textual representations of gene pairs

**DOI:** 10.1093/bioadv/vbag140

**Published:** 2026-05-21

**Authors:** Rida Assaf, Basel Fakhri

**Affiliations:** Department of Computer Science, American University of Beirut, Riad El-Solh 1107 2020, Lebanon; Department of Computer Science, American University of Beirut, Riad El-Solh 1107 2020, Lebanon

## Abstract

**Motivation:**

Operons are fundamental units of gene regulation in bacteria and can provide valuable insights into genome organization, co-expression, and functional relationships between genes. Computational prediction of operons can support downstream analyses such as pathway reconstruction, comparative genomics, and gene function inference. However, many existing tools rely on rigid feature engineering or curated interaction networks, limiting scalability and applicability to poorly annotated genomes.

**Results:**

We propose a transformer-based approach that reformulates operon prediction as a binary text classification task over adjacent gene pairs. By serializing genomic features, including gene orientation, intergenic distance, GC content, functional annotations, protein families, and conservation, into natural language descriptions, we enable pre-trained language models to perform operon classification using flexible, widely available inputs. A RoBERTa-based model achieves competitive predictive performance under multiple evaluation settings, including leave-one-species-out analysis over six bacterial genomes and benchmark comparisons on standard datasets. Through ablation and inference-time resilience analyses, we demonstrate that sequence-derived and annotation-based features are sufficient for competitive performance, and that performance remains stable when selected features are removed at test time.

**Availability and implementation:**

All datasets required to reproduce this study are publicly available at GitHub repository. Genome annotations were retrieved through the BV-BRC platform.

## 1 Introduction

In bacterial genomes, operons are functional clusters of genes that are transcribed together under the control of a single promoter and terminator. The genes within an operon often encode proteins involved in related biological processes, such as metabolic pathways, stress responses, or virulence. Since their discovery in the early 1960s (Fran *et al.* 1960), operons have provided critical insights into the mechanisms of gene regulation. Accurately predicting operons enhances gene function annotation via label propagation (Joon *et al.* 2010), supports regulatory network reconstruction, and informs applications in drug target discovery and antibiotic resistance analysis (Pantosti *et al.* 2007, Wang *et al.* 2007).

A wide range of computational approaches have been developed for operon prediction, evolving from rule-based heuristics to modern machine learning techniques. Early methods primarily relied on intergenic distance, gene orientation, and simple statistical thresholds (Romero and Karp 2004). Subsequent models incorporated gene expression profiles, conservation metrics, and curated genomic features to train supervised classifiers (Taboada *et al.* 2010, Fortino *et al.* 2014). Databases such as DOOR2 (Mao *et al.* 2014) and ProOpDB (Taboada *et al.* 2012) emerged to provide operon annotations and predictions based on these structured features. However, the availability and scalability of such features poses challenges for broad applicability and scalability.

More recent deep learning approaches, including our own Operon Hunter (Assaf *et al.* 2021), represent gene neighborhoods as images and apply convolutional neural networks (CNNs) for operon prediction. While Operon Hunter achieves high predictive performance, it incurs significant computational overhead due to the preprocessing required to extract pileup-based features and generate images. This limits its scalability to large or fragmented genomic datasets. A derivative tool, Operon Finder (Tomar *et al.* 2023), is based on Operon Hunter but inherits the same scalability challenges. Other models have focused on promoter and terminator detection using Hidden Markov Models (HMMs) (Yada *et al.* 1999, Craven *et al.* 2000, Tjaden *et al.* 2002), or employed artificial neural networks trained on STRING-derived functional associations (Szklarczyk *et al.* 2017, Zaidi and Zhang 2017). Despite their success, many of these methods rely on either species-specific structured features or curated interaction networks such as STRING, limiting their applicability to less-annotated genomes and potentially introducing indirect information leakage through reliance on *post hoc* curated interaction databases.

Recent advances in representation learning suggest that heterogeneous structured data can be reformulated as natural language, allowing transformer architectures to model complex relationships within a unified descriptive framework.

In this work, we introduce a novel approach to operon prediction that reframes gene-pair classification as a natural language processing task. By serializing genomic features into textual descriptions, we enable transformer-based language models to make operon predictions without requiring rigid feature engineering pipelines. Our formulation prioritizes features that are either sequence-derived (e.g. gene orientation, intergenic distance, GC content) or easily obtainable from large-scale annotation platforms such as the Bacterial and Viral Bioinformatics Resource Center (BV-BRC) (Olson *et al.* 2023) (e.g. protein family assignments, functional annotations, and conservation metrics).

A central design principle of our approach is to avoid reliance on difficult-to-obtain signals such as expression profiles or curated interaction networks. This enables broad applicability to genomes with limited annotation. When available, features like STRING co-function scores may offer a modest benefit, but we show that the model performs robustly even in their absence. Through ablation and feature removal experiments, we demonstrate that performance remains resilient when key features are missing at test time, highlighting the model’s suitability for real-world genomic settings with incomplete or noisy data.

To our knowledge, this is the first operon prediction method to leverage large language models and textual serialization of genomic features. While transformers have shown utility in protein modeling and regulatory sequence analysis (Rao *et al.* 2021, Tang *et al.* 2025), their application to structural genome annotation tasks like operon prediction remains largely unexplored. We evaluate our method under multiple experimental regimes—including leave-one-species-out (LOSO), and cross-species setups—and show that it achieves competitive performance on benchmark genomes while supporting broader applicability within bacterial genomes through its reliance on minimally engineered, widely available features.

## 2 Methods

### 2.1 Task formulation and input representation

We frame operon prediction as a binary classification task over adjacent gene pairs: given two neighboring genes within a bacterial genome, the goal is to predict whether they belong to the same operon. This formulation aligns with prior work in operon prediction (Taboada *et al.* 2012, Mao *et al.* 2014, Assaf *et al.* 2021), which commonly treats operon inference as a gene-pair classification problem.

Rather than using fixed-format feature vectors, we represent each gene pair as a paragraph of natural language text that encodes relevant genomic features. This reformulation enables the use of pre-trained language models for classification while decoupling the model from rigid feature schemas.

**Figure 1:**
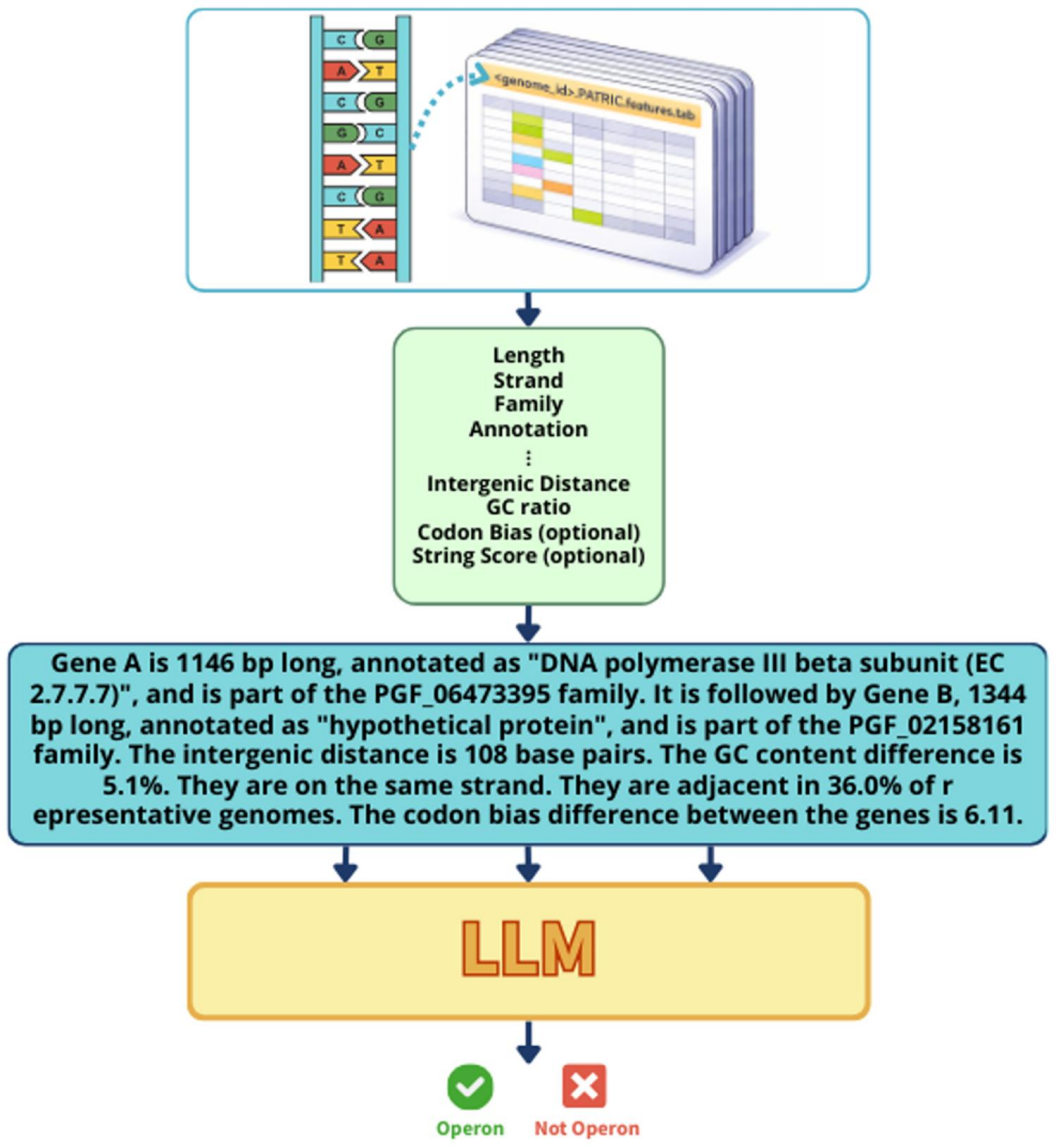
Overview of the operon prediction framework. Genome sequences and annotation files are used to extract sequence-derived and annotation-based features for adjacent gene pairs, including gene length, strand orientation, intergenic distance, GC content difference, protein family assignments, functional annotations, and conservation metrics. These structured features are serialized into natural language descriptions, forming a textual representation of each gene pair. The serialized text is then provided to a fine-tuned transformer model (RoBERTa) for binary classification, producing an operon or non-operon prediction. Optional features such as STRING interaction scores or codon bias can be incorporated when available.

Figure 1 illustrates the full pipeline: from sequence-derived and annotation-based features to serialized text to transformer-based classification.

### 2.2 Feature design and textual encoding

Each gene pair is transformed into a structured paragraph capturing the following features:

- Gene lengths
- Intergenic distance
- Strand orientation
- GC content difference
- Functional annotations
- Protein family identifiers
- Adjacency conservation scores across reference genomes

Each adjacent gene pair is converted into a fixed natural-language description using a deterministic template. The template encodes sequence-derived and annotation-derived features in a structured sentence format. Specifically, each instance is serialized as:

*“Gene A is {length_A} bp long, annotated as ‘{function_A}’, and is part of the {family_A} family. It is followed by Gene B, {length_B} bp long, annotated as ‘{function_B}’, and is part of the {family_B} family. The intergenic distance is {distance} base pairs. The GC content difference is {gc_diff}%. They are on the {strand_orientation} strand. They are adjacent in {adjacency_percent}% of representative genomes.”*

Numerical values are inserted directly without discretization. Categorical variables (e.g. strand orientation) are rendered as literal text (e.g. “same strand” or “opposite strands”). Features excluded in specific ablation experiments are omitted deterministically from the template. The full serialization scripts are available in the Code availability repository. Protein families were assigned using PGFAMs (Davis *et al.* 2016), a hierarchical system of protein family identifiers developed as part of the PATRIC (Wattam *et al.* 2017) and BV-BRC annotation pipelines. These identifiers group homologous genes across bacterial genomes and enable functional comparisons at the family level.

The method operates on genome annotations containing gene coordinates, strand information, and functional descriptors. In this study, annotations were obtained from BV-BRC in tabular (PATRIC.features.tab) format. Sequence-derived features, including: gene length, intergenic distance, and strand orientation, were computed from the annotation data, while GC content differences were computed directly from the corresponding genome sequence files (FASTA format). Additional features, including functional annotations, and adjacency conservation scores, were incorporated when available. While this work uses BV-BRC-derived annotation and sequence files, the approach is not restricted to a specific format, provided equivalent gene-level information and genome sequences are available.

Adjacency conservation was computed by scanning a curated set of ∼16 000 representative bacterial genomes from the BV-BRC database. For a given gene pair, we calculated the percentage of genomes in which a homologous pair (based on PGFAM membership) appeared adjacent. This yielded a conservation score ranging from 0% to 100% and captures evolutionary co-localization across taxa. The conservation score was computed independently of operon labels and reflects only adjacency frequency at the PGFAM family level. Among the six genomes used for model development, only three were present in the broader 16 000 genome reference set used to estimate conservation, and their contribution represents a negligible fraction of the total reference collection. Consequently, conservation values encode global evolutionary adjacency patterns rather than species-specific operon annotations, mitigating the risk of information leakage during cross-species evaluation. While a stricter exclusion-based recomputation of conservation features could be considered, the negligible overlap and unsupervised nature of this feature make meaningful information leakage unlikely.

When available, STRING co-function scores were appended as additional sentences to indicate predicted functional associations derived from curated interaction networks. STRING integrates heterogeneous evidence sources, including experimental data, co-expression patterns, orthology relationships, gene neighborhood signals, and literature mining. To assess the model’s dependence on such curated resources and to mitigate potential indirect information leakage from *post hoc* knowledge bases, we conducted experiments both with and without STRING features. This design allows us to disentangle predictive signal derived from intrinsic genomic features versus externally curated interaction networks.

We note that some other features (e.g. functional annotation, conservation) may also be unavailable in some genomes. Accordingly, we performed ablation and resilience analyses to evaluate model performance when such features are systematically excluded either during training or at inference time. These experiments are described in Section 3 and help characterize the flexibility of the textual formulation in real-world, annotation-variable settings.

### 2.3 Dataset construction

We constructed a labeled dataset of adjacent gene pairs for operon prediction using annotated bacterial genomes from the BV-BRC database using the genome identifiers listed in Table 1. Each instance consists of a pair of neighboring genes, labeled as positive if both genes belong to the same operon and negative otherwise. Because the model operates on adjacent gene pairs, dataset composition is reported at the gene-pair level. Labels were derived from trusted databases as described below.

**Table 1:** Compostition of the ODB-derived operon gene-pair dataset across six bacterial genomes.

Genome Name	BV-BRC genome ID	Total gene pairs	Positive (operonic)	Negative (non-operonic)
*Escherichia coli* str. K-12 substr. MG1655	511 145.12	2765	1443	1322
*Listeria monocytogenes* EGD-e	169 963.11	1584	806	778
Legionella pneumophila str. Paris	297 246.15	1402	611	791
Corynebacterium glutamicum ATCC 13032	196 627.14	919	524	395
*Bacillus subtilis* subsp. Subtilis str. 168	224 308.43	913	474	439
*Photobacterium profundum* SS9	298 386.8	885	447	438

Number of adjacent gene pairs labeled as operonic (positive) or non-operonic (negative) based on annotations from the Known Operons section of the Operon DataBase (ODB). These gene pairs constitute the full dataset used for LOSO and mixed-species training and evaluation.

For model development, we adopted the strategy used in OperonHunter (Assaf *et al.* 2021), leveraging high-confidence operons from the Known Operons section of the Operon DataBase (ODB) (Okuda and Yoshizawa 2011). Six genomes with the highest number of annotated operons were selected. Across the six training genomes, the dataset contains a total of 8468 gene pairs, comprising 4305 positive (50.8%) and 4163 negative (49.2%) instances. Table 1 summarizes the dataset composition per genome. The selected genomes span multiple bacterial phyla, including *Bacillota*, *Actinomycetota*, and *Pseudomonadota*, and include both Gram-positive and Gram-negative organisms. Most genomes exhibit near-balanced class proportions (around 49%–51% operonic), with only moderate deviation in two genomes (44%–57% operonic).

Positive (operonic) gene pairs were drawn exclusively from multi-gene operons. Negative (non-operonic) examples were constructed by pairing the terminal gene of each annotated operon with its immediately adjacent downstream neighbor outside the operon boundary. This ensures that negative examples consist of genomically adjacent gene pairs with realistic contextual similarity, avoiding artificially distant or trivial negatives. This strategy follows established conventions in prior operon prediction studies (Taboada *et al.* 2012).

For benchmarking against prior operon prediction tools, we constructed additional test sets for *Escherichia coli* and *Bacillus subtilis*. These genomes are widely used in operon prediction studies due to the availability of experimentally validated operons. For each genome, we selected operons that were independently confirmed in both ODB and the genome-specific validated operon database: RegulonDB for *E. coli* (Tierrafría *et al.* 2022), and DBTBS for *B. subtilis* (Sierro *et al.* 2008). This intersection ensures high-confidence operonic labels. The corresponding non-operonic pairs were again selected at operon boundaries, requiring presence in any of the referenced databases. The resulting benchmark datasets contain 822 gene pairs for *E. coli* (361 operonic, 461 non-operonic) and 671 gene pairs for *B. subtilis* (369 operonic, 302 non-operonic). To ensure direct comparability with prior work, including OperonHunter (Assaf *et al.* 2021), we followed the same benchmark construction protocol and dataset definitions. These benchmark datasets were used exclusively for comparative evaluation.

Model performance was evaluated under two protocols. In the LOSO setting, one genome was held out entirely for testing while the model was trained on gene pairs from the remaining five genomes. Within each training fold, 10% of the training data were reserved for validation, and no gene pairs from the held-out genome were used during training or model selection. As a complementary evaluation, gene pairs from all six genomes were pooled and randomly partitioned into 80% training, 10% validation, and 10% testing sets. This mixed-species setting does not enforce genome-level separation and serves as a conventional machine learning baseline.

All datasets, preprocessing scripts, and code used to generate gene-pair representations are publicly available at the repository link provided in the Data availability section.

### 2.4 Dataset construction

We fine-tuned the pre-trained RoBERTa-base transformer model (Liu *et al.* 2019) using the HuggingFace transformers library implemented in PyTorch. RoBERTa-base consists of 12 transformer encoder layers, 768 hidden dimensions, and 12 self-attention heads per layer (∼125 million parameters).

Full fine-tuning was performed, updating all transformer layers during training rather than freezing any subset of weights. This allows the model’s contextual representations to adapt to the structured genomic descriptions used in our task. Each gene-pair description was tokenized using RoBERTa’s byte-level byte-pair encoding (BPE) tokenizer. We retained the model’s default maximum sequence length of 512 tokens. To evaluate whether this limit affected our inputs, we analyzed the tokenized length distribution across all datasets. The longest observed sequence was 324 tokens, well below the 512-token threshold. Consequently, no input sequences were truncated during training or evaluation, and padding was applied only to shorter sequences for batching purposes.

Binary operon classification was performed using a sequence classification head on top of the encoder. The model was trained using the AdamW optimizer with the following hyperparameters:

- Learning rate: 3 × 10^−5^
- Weight decay: 0.01
- Batch size: 32
- Learning rate scheduler: linear decay
- Maximum training epochs: 10

Binary cross-entropy loss was minimized for the classification objective.

Model evaluation and checkpointing were performed at the end of each epoch (evaluation_strategy = “epoch”; save_strategy = “epoch”). Although training was configured for 10 epochs, validation performance consistently converged after ∼3 epochs across all datasets. The final model was selected as the checkpoint achieving the best validation performance.

All experiments were conducted using a fixed random seed to ensure reproducibility. Training was performed on an NVIDIA A100 GPU, with an average training time of ∼7 minutes per run, depending slightly on dataset size and split configuration. All experiments were conducted using Python 3.12.13, PyTorch 2.10, HuggingFace Transformers 5.0, Pandas 2.2.2, Numpy 2.0.2, Datasets 4.0, and scikit-learn 1.6.

All preprocessing pipelines, training scripts, configuration files, and pretrained model checkpoints required to reproduce these experiments are publicly available (see Data availability section).

## 3 Results

We evaluated our operon prediction framework across multiple experimental settings to assess (i) cross-species generalization, (ii) comparative performance against existing tools, (iii) contribution of individual feature groups, and (iv) robustness to missing annotations.

### 3.1 Cross-species generalization (LOSO)

In the LOSO setting, one genome was held out entirely for testing while the model was trained on gene pairs from the remaining five genomes. Within each fold, 10% of the training data was reserved for validation, and no data from the held-out genome were used during training or model selection. This protocol enforces strict genome-level separation and provides a rigorous assessment of cross-species generalization.

The reported LOSO results correspond to the model trained without STRING features. Under this configuration, the model achieved consistent performance across the six genomes, with accuracy ranging from 71.5% to 88.1% and PR-AUC values between 79.2% and 91.7% (Table 2). Sensitivity and specificity remained well-balanced in most genomes, although variation in sensitivity was observed for certain species. Overall, performance trends indicate effective generalization across phylogenetically distinct bacteria spanning *Bacillota*, *Actinomycetota*, and *Pseudomonadota*. Feature contribution analyses are presented below.

**Table 2:** Gene-pair classification performance under leave-one-specie (LOSO) evaluation across six bacterial genomes.^a^

Genome	Sensitivity (%)	Specificity (%)	F1-score (%)	PR AUC (%)	Accuracy (%)
*Escherichia coli*	88.57	85.40	87.71	90.71	87.05
*Listeria monocytogenes*	85.36	90.86	87.92	91.73	88.06
*Legionella pneumophila*	82.65	82.05	80.29	84.13	82.31
*Corynebacterium glutamicum*	75.43	90.56	82.67	90.48	81.90
*Bacillus subtilis*	89.03	86.31	88.10	90.91	87.70
*Photobacterium profundum*	61.52	81.74	68.58	79.21	71.53

a Performance metrics under a mixed-species 80-10-10 train-validation-test split, in which gene pairs from all six genomes were pooled and randomly partitioned. Feature sets range from sequence-derived baseline features to progressively enriched combinations including conservation, functional annotation, protein family, and STRING interaction features. This setting complements the primary leave-one-species-out evaluation by assessing performance without genome-level separation.

### 3.2 Benchmark comparison on standard operon datasets

For comparability with prior operon prediction studies, we evaluated performance on *E. coli* and *B. subtilis* using benchmark test sets constructed from operons independently confirmed in both ODB and genome-specific validated operon databases (RegulonDB for *E. coli* and DBTBS for *B. subtilis*). These genomes are widely used in the literature and allow direct comparison with existing tools.

In this benchmarking setting, models were trained under strict LOSO conditions, ensuring that the target genome was excluded entirely from training. The feature configuration used for benchmarking included sequence derived, conservation, functional annotation, and protein family features, without incorporating STRING interaction scores.

Comparative performance against existing tools is summarized in Table 3. Across both *E. coli* and *B. subtilis*, the model achieves competitive sensitivity and specificity relative to established operon prediction tools. Notably, this performance is obtained while maintaining strict genome-level separation during training, and without reliance on curated interaction networks, demonstrating that structured genomic features provide sufficient signal for competitive operon prediction under strict genome-level separation. Corresponding confusion matrices for the benchmark evaluations are provided in Figs. 1 and 2, available as supplementary data at *Bioinformatics Advances* online.

**Table 3:** Comparison of operon prediction tools on benchmark datasets for *Escherichia coli* and *Bacillus subtilis*.^a^

Tool	*E. coli*		*B. subtilis*		Acc. (%)	Sens. (%)	Prec. (%)	F1 (%)
	Sens. (%)	Spec. (%)	Sens. (%)	Spec. (%)				
OperonHunter	88	95	97	88	92.0	92.5	92.0	92.1
ProOpDB	93	90	93	88	91.0	93.0	89.0	90.8
DOOR	81	94	86	97	89.0	83.5	94.0	88.6
Transformer (ours)	87.5	89.4	93.8	86.7	89.4	90.4	88.0	89.1

a Performance comparison against established operon prediction tools on benchmark test sets constructed from operons independently confirmed in ODB and genome-specific validated databases (RegulonDB for *E. coli* and DBTBS for *B. subtilis*). Results for the transformer model reflect strict leave-one-species-out (LOSO) training with sequence-derived, conservation, functional, and protein family features (no STRING).

### 3.3 Feature contribution analysis

To quantify the contribution of different feature categories, we conducted ablation analyses under both LOSO and mixed-species settings.

Under LOSO evaluation, progressively enriched feature sets were assessed beginning with a minimal sequence-derived baseline representation (gene length, intergenic distance, GC content difference, and strand orientation), and incrementally adding conservation, functional annotation, protein family, and STRING features. The baseline configuration already achieved strong performance across most genomes, reflecting the predictive power of structural genomic features. The addition of contextual and evolutionary features produced modest and genome-dependent changes in performance. In several species (e.g. *E. coli*, *B. subtilis*, and *Listeria monocytogenes*), enriched feature sets yielded small improvements in F1-score and PR-AUC, whereas in others the gains were limited or reflected shifts in sensitivity-specificity balance rather than uniform improvement. Inclusion of STRING interaction scores provided incremental benefit in certain cases but did not uniformly dominate non-STRING configurations. Detailed per-genome ablation results under the LOSO setting are provided in Tables 1–6, available as supplementary data at *Bioinformatics Advances* online.

**Table 4:** Performance under different feature combinations (mixed-species 80-10-10 train-validation-test split).^a^

Feature det	Accuracy (%)	Sensitivity (%)	Specificity (%)	F1-score (%)	___PR AUC (%)
Baseline	83.73	79.35	88.25	83.21	88.66
+Function	83.84	82.83	84.89	83.90	88.28
+Conservation	84.43	82.60	86.33	84.36	88.82
+Function + Conservation	85.26	81.90	88.73	84.96	89.68
+Function + Conservation + Family	83.96	83.99	83.93	84.18	88.25
+Function + Conservation + Family + String	84.67	80.51	88.97	84.22	89.36

a Performance metrics under a mixed-species 80-10-10 train-validation-test split, in which gene pairs from all six genomes were pooled and randomly partitioned. Feature sets range from sequence-derived baseline features to progressively enriched combinations including conservation, functional annotation, protein family, and STRING interaction features. This setting complements the primary leave-one-species-out evaluation by assessing performance without genome-level separation.

**Table 5:** Performance under inference-time feature removal in the LOSO setting.^a^

	*E. coli*					*B. subtilis*				
Feature removed	Accuracy (%)	Sensitivity (%)	Specificity (%)	F1-score (%)	PR AUC (%)	Accuracy (%)	Sensitivity (%)	Specificity (%)	F1-score (%)	PR AUC (%)
None	88.56	87.53	89.37	87.05	89.79	90.46	93.77	86.42	91.53	93.30
Family	87.83	87.81	87.85	86.38	89.08	91.36	93.50	88.74	92.25	94.05
Function, family	84.18	86.98	82.00	82.85	85.90	90.76	94.58	86.09	91.84	93.41
Function, family, conservation	85.64	86.15	85.25	84.05	87.15	90.91	94.31	86.75	91.94	93.56

a Performance metrics (accuracy, sensitivity, specificity, F1-score, and PR-AUC) for *Escherichia coli* and *Bacillus subtilis* under sequential removal of contextual features at inference time. All models were trained without STRING. Feature removal was applied only at evaluation, while training used the full non-STRING feature configuration.

We additionally performed a complementary ablation study under a mixed-species 80-10-10 train-validation-test split, in which gene pairs from all six genomes were pooled and randomly partitioned. This setting provides a conventional machine learning baseline without genome-level separation. Results are summarized in Table 4.

The baseline model using only sequence-derived structural features already achieved strong performance (F1 = 83.2%, PR-AUC = 88.7%), confirming their predictive value. The addition of functional and conservation features yielded modest improvements in F1-score and PR-AUC, with the combination of function and conservation producing the strongest overall performance in this setting. Inclusion of protein family features did not consistently improve results, and adding STRING interaction scores primarily shifted the sensitivity-specificity balance rather than substantially increasing overall F1-score.

Overall, feature enrichment provided incremental, configuration-dependent gains rather than dramatic performance shifts, consistent with trends observed under LOSO evaluation.

### 3.4 Robustness to missing annotations (inference-time resilience)

To assess robustness in settings where annotations may be incomplete, we evaluated inference-time feature removal. We performed resilience experiments using a model trained without STRING because, in preliminary tests, models trained with STRING failed to generalize when STRING was removed and experienced severe degradation in performance, approaching random-level classification in some settings. This suggests that models trained with STRING incorporate this strong interaction signal into their decision boundary, and performance degrades when the feature is removed at inference due to feature distribution shift. This behavior reflects reliance on the presence of a strong feature during training rather than lack of signal in baseline features. Using a model trained on all features except STRING, we sequentially removed function, family, and conservation features at inference. Results are summarized in Table 5.

Across both *E. coli* and *B. subtilis*, performance remained stable as contextual features were sequentially removed at inference time. In *E. coli*, removal of functional and conservation features led to modest shifts in specificity and F1-score, while sensitivity changed only slightly. In *B. subtilis*, sensitivity remained consistently high across all reduced-feature configurations, with only minor variation in specificity and PR-AUC.

These results indicate that while contextual and evolutionary features provide incremental improvements in certain settings, the model retains strong predictive capacity even when limited to structural genomic features. Overall, the framework demonstrates resilience to incomplete annotation coverage without substantial performance degradation.

## 4 Discussion

We present a transformer-based approach to operon prediction that reframes gene-pair classification as a language modeling task. By serializing genomic features into structured textual descriptions, we enable pre-trained language models to leverage linguistic representations of biological data, a departure from traditional feature vector pipelines. This work evaluates whether structured genomic attributes, when expressed as text, can be effectively modeled using transformer architectures. In this sense, the study serves as a methodological framework with empirical validation in operon prediction.

Our results demonstrate that transformer models fine-tuned on these textual representations achieve competitive performance across bacterial genomes. In LOSO evaluation across all six genomes, the model achieved balanced sensitivity and specificity across phylogenetically distinct organisms spanning *Bacillota*, *Actinomycetota*, and *Pseudomonadota*, indicating generalization within the evaluated bacterial diversity. For comparability with prior literature, we additionally report benchmark results on *B. subtilis* and *E. coli*, two widely used operon evaluation genomes. The consistency of performance across held-out genomes suggests that the learned decision boundary captures broadly transferable genomic patterns rather than relying solely on genome-specific heuristics.

Nevertheless, evaluation remains limited to bacterial genomes with curated operon annotations, and broader testing across additional taxa will be necessary to assess the scope of generalization beyond this domain.

A key contribution of this work is demonstrating that strong predictive performance can be achieved using features that are either sequence-derived or readily available from standard annotation pipelines such as BV-BRC. Our ablation and resilience experiments show that gene function, protein family, strand orientation, intergenic distance, and conservation provide sufficient signal for competitive classification performance across evaluated genomes. Although STRING-derived interaction scores can improve performance slightly, the model remains effective without them, supporting applicability without reliance on curated interaction networks. Notably, models trained with STRING features exhibited a pronounced drop in performance when these features were removed at inference time. When STRING features are included during training, the model naturally incorporates this strong interaction signal into its decision boundary. Removing it at inference introduces distribution shift, leading to reduced sensitivity. The strong degradation observed when STRING-based features are removed at inference time suggests that models trained with these features may become highly dependent on curated interaction signals. STRING encodes functional associations derived from experimental data, co-expression, and orthology, which are strongly correlated with operon structure. When such features are available during training but absent during inference, the learned decision boundary may fail to compensate for their removal, leading to severe performance collapse.

In contrast, resilience experiments were conducted using models trained without STRING. Under this setting, sequential removal of functional, family, and conservation features led to gradual performance degradation rather than collapse. This pattern suggests that contextual and evolutionary features help reduce false negatives and stabilize operon detection. When only structural features are available, the model appears to adopt a more conservative prediction profile, resulting in lower sensitivity but preserved specificity.

Annotation coverage varies substantially across bacterial genomes, particularly for functional annotations and curated interaction networks. Well-studied organisms such as *E. coli* benefit from dense functional characterization, whereas many genomes lack comparable resources. Our inference-time ablation experiments explicitly simulate such reduced-annotation settings by removing functional, or conservation features. The model retains competitive performance under these constraints, suggesting robustness to uneven annotation availability across organisms.

The serialization of structured genomic features into natural language offers representational flexibility. Conceptually aligned with prior work on textual serialization of tabular data for transformer-based modeling (Hegselmann *et al.* 2023), this approach expresses numerical (e.g. intergenic distance), categorical (e.g. strand orientation), relational (e.g. interaction scores), and semantic (e.g. functional annotations) attributes within a shared descriptive format. Rather than constructing task-specific encodings for each feature type, the model receives a structured narrative of gene context.

Textual representations allow transformers to exploit semantic similarity in functional annotations, enabling generalization beyond rigid categorical encodings. Because functional descriptions are expressed in natural language rather than as isolated identifiers, lexical and contextual similarity between related gene functions may be leveraged implicitly.

However, this approach also has limitations. Transformer models introduce greater computational overhead compared to lightweight classifiers, and their expressive capacity must be balanced against efficiency considerations. Moreover, although the architecture is scalable, our evaluation is currently limited to six bacterial genomes with significant curated operon annotations. Broader validation across additional bacterial clades, as well as exploration in phylogenetically distant organisms, would further clarify the scope of generalization.

Because the framework operates at the adjacent gene-pair level, reconstruction of multi-gene operons requires aggregating consecutive positive predictions. While pairwise evaluation is standard in operon prediction literature and enables direct comparison with prior methods, local misclassifications may propagate when inferring complete operon boundaries.

In addition, operon organization is not strictly binary in biological systems. Alternative promoters and terminators can generate condition-specific transcriptional units, such that adjacent genes may be co-transcribed under some conditions but not others. As in prior computational operon prediction studies, the present work adopts a static binary formulation based on curated operon annotations. This abstraction enables standardized benchmarking but does not capture the full regulatory complexity of bacterial transcription.

The proposed serialization framework may be extended in several directions. First, span-based or sequence-to-sequence formulations could model complete operon boundaries directly rather than aggregating pairwise predictions. Second, qualitative analysis of attention patterns may provide insight into which serialized features most strongly influence individual predictions, offering an avenue for interpretability. Finally, we chose the Roberta-base model due to its strong performance on general-domain classification tasks and its robust handling of syntactically rich text. Unlike generative models, RoBERTa is encoder-only and optimized for sequence classification, making it well-suited for the binary decision task we formulate. Although input lengths did not approach the 512-token limit in this study, longer-context transformer architectures could facilitate incorporation of promoter regions or extended genomic context in future work. These directions remain exploratory and warrant systematic evaluation.

This work does not aim to replace lightweight operon predictors optimized for efficiency. Rather, it illustrates that structured genomic prediction tasks can be reformulated within a textual transformer framework, allowing heterogeneous biological signals to be expressed within a unified descriptive representation. Although classical features such as strand orientation and intergenic distance already capture strong predictive signal, the serialization approach offers a flexible abstraction that can incorporate additional contextual or semantic information without redesigning fixed feature matrices. Such flexibility may become increasingly relevant as annotation resources expand and foundation models continue to evolve.

Overall, we position this study not as a replacement for established operon prediction tools, but as a methodological exploration of representational strategy. The results indicate that structured genomic attributes can be effectively modeled within a textual transformer formulation, providing a complementary framework that remains robust across varying levels of annotation availability.

## 5 Conclusion

We presented a transformer-based framework for operon prediction that reformulates gene-pair classification as a textual modeling task. By serializing genomic features into structured natural language descriptions, the approach enables transformer architectures to operate on heterogeneous genomic attributes without requiring fixed tabular encodings or curated interaction networks.

The framework prioritizes features that are either directly sequence-derived, such as intergenic distance, strand orientation, and GC content, or readily obtainable from large-scale annotation platforms like BV-BRC, including functional annotations and protein family assignments. While additional features like STRING-based functional associations can be optionally incorporated, our results indicate that competitive performance can be achieved without reliance on curated interaction networks.

Across multiple evaluation regimes, including LOSO analysis, the model demonstrates consistent performance within the evaluated bacterial diversity and retains stability when selected features are removed at inference time. Overall, we position this work as a flexible representational strategy for operon prediction in annotation-variable bacterial genome settings, illustrating how transformer models can be adapted for structured genome annotation tasks through domain-aware textual representations.

## Supplementary Material

vbag140_Supplementary_Data

## Data Availability

All datasets required to reproduce this study are publicly available at: https://github.com/Core-Aub/Operons_LLMs. All genome annotations were originally retrieved via the BV-BRC platform (Olson *et al.* 2023). The redistributed files are limited to those necessary for reproducibility and comply with BV-BRC data access policies.
