# Supplementary material for: Transformer-based operon prediction using textual representations of gene pairs
Source: Bioinform Adv. 2026 May 21;6(1):vbag140. doi: 10.1093/bioadv/vbag140 (PMC13221980; doi:10.1093/bioadv/vbag140)

**Supplementary Material**

**Supplementary Information for:**

**Transformer-Based Operon Prediction Using Textual Representations of Gene Pairs**

Authors: Rida Assaf and Basel Fakhri

**Supplementary Tables**

Table S1. LOSO feature ablation – *Listeria monocytogenes*

| **Feature Set** |  | *Listeria monocytogenes* | | |  |
| --- | --- | --- | --- | --- | --- |
|  | Accuracy | Sensitivity | Specificity | F1-score | PR AUC |
| Vanilla only | 88.12% | **90.69%** | 85.46% | 88.61% | 91.02% |
| + Conservation | 88.63% | 89.33% | 87.90% | 88.89% | 91.61% |
| + Function (no cons.) | 89.13% | 88.21% | 90.09% | 89.21% | 92.21% |
| + Function + Cons. | 89.45% | 88.96% | 89.96% | 89.57% | 92.39% |
| + Function + Family + Cons. | 88.06% | 85.36% | 90.86% | 87.92% | 91.73% |
| + STRING (Full) | **90.46%** | 86.72% | **94.34%** | **90.25%** | **93.78%** |

Table S2. LOSO feature ablation – *Corynebacterium glutamicum*

| **Feature Set** |  | *Corynebacterium glutamicum* | | |  |
| --- | --- | --- | --- | --- | --- |
|  | Accuracy | Sensitivity | Specificity | F1-score | PR AUC |
| Vanilla only | 82.88% | 76.57% | 91.33% | 83.66% | 91.09% |
| + Conservation | 83.97% | 78.86% | 90.82% | 84.92% | 91.48% |
| + Function (no cons.) | 83.53% | 77.14% | 92.09% | 84.29% | 91.56% |
| + Function + Cons. | 79.83% | 69.71% | **93.37%** | 79.83% | 90.21% |
| + Function + Family + Cons. | 81.90% | 75.43% | 90.56% | 82.67% | 90.48% |
| + STRING (Full) | **85.17%** | **79.81%** | 92.35% | **86.04%** | **92.34%** |

Table S3. LOSO feature ablation – *Legionella pneumophila*

| **Feature Set** |  | *Legionella pneumophila* | | |  |
| --- | --- | --- | --- | --- | --- |
|  | Accuracy | Sensitivity | Specificity | F1-score | PR AUC |
| Vanilla only | 83.95% | 84.45% | 83.57% | 82.10% | 85.55% |
| + Conservation | 83.10% | 78.72% | 86.47% | 80.23% | 84.90% |
| + Function (no cons.) | **84.02%** | **85.27%** | 83.05% | **82.31%** | **85.62%** |
| + Function + Cons. | 82.17% | 74.47% | **88.12%** | 78.45% | 84.24% |
| + Function + Family + Cons. | 82.31% | 82.65% | 82.05% | 80.29% | 84.13% |
| + STRING (Full) | 83.24% | 82.16% | 84.07% | 81.03% | 84.94% |

Table S4. LOSO feature ablation – *Photobacterium profundum*

| **Feature Set** |  | *Photobacterium profundum* | | |  |
| --- | --- | --- | --- | --- | --- |
|  | Accuracy | Sensitivity | Specificity | F1-score | PR AUC |
| Vanilla only | 72.09% | 63.76% | 80.59% | **69.77%** | 79.55% |
| + Conservation | **72.32%** | 60.63% | **84.25%** | 68.87% | 80.11% |
| + Function (no cons.) | 71.98% | 62.86% | 81.28% | 69.38% | 79.51% |
| + Function + Cons. | 71.41% | **64.88%** | 78.08% | 69.63% | 78.87% |
| + Function + Family + Cons. | 71.53% | 61.52% | 81.74% | 68.58% | 79.21% |
| + STRING (Full) | 71.77% | 61.74% | 84.02% | 69.61% | **80.42%** |

Table S5. LOSO feature ablation – *Escherichia coli*

| **Feature Set** |  | *Escherichia Coli* | | |  |
| --- | --- | --- | --- | --- | --- |
|  | Accuracy | Sensitivity | Specificity | F1-score | PR AUC |
| Vanilla only | 85.64% | 86.28% | 84.95% | 86.25% | 89.83% |
| + Conservation | 86.11% | **91.48%** | 80.26% | 87.30% | 89.71% |
| + Function (no cons.) | 85.64% | 85.86% | 85.40% | 86.19% | 89.88% |
| + Function + Cons. | 86.29% | 89.40% | 82.90% | 87.19% | 90.01% |
| + Function + Family + Cons. | 87.05% | 88.57% | 85.40% | 87.71% | 90.71% |
| + STRING (Full) | **87.59%** | 88.43% | **86.46%** | **88.06%** | **91.08%** |

Table S6. LOSO feature ablation – *Bacillus subtilis*

| **Feature Set** |  | *Bacillus Subtilis* | | |  |
| --- | --- | --- | --- | --- | --- |
|  | Accuracy | Sensitivity | Specificity | F1-score | PR AUC |
| Vanilla only | 87.59% | 85.23% | **90.07%** | 87.54% | 91.38% |
| + Conservation | 86.08% | **90.51%** | 81.46% | 86.93% | 89.49% |
| + Function (no cons.) | 87.92% | 88.61% | 87.20% | 88.24% | 91.15% |
| + Function + Cons. | 87.27% | 89.24% | 85.21% | 87.76% | 90.53% |
| + Function + Family + Cons. | 87.70% | 89.03% | 86.31% | 88.10% | 90.91% |
| + STRING (Full) | **88.46%** | 88.61% | 88.30% | **88.70%** | **91.61%** |

**Supplementary Figures**

**Figure S1. Confusion matrices for Bacillus subtilis under leave-one-species-out (LOSO) evaluation across progressively enriched feature configurations.**

Panels correspond to the following feature sets:

(a) sequence-derived baseline features (gene length, intergenic distance, GC content difference, strand orientation);

(b) baseline + conservation;

(c) baseline + functional annotation;

(d) baseline + conservation + functional annotation;

(e) baseline + conservation + functional annotation + protein family;

(f) baseline + conservation + functional annotation + protein family + STRING interaction score.

Rows indicate true labels (0 = non-operonic, 1 = operonic), and columns indicate predicted labels.


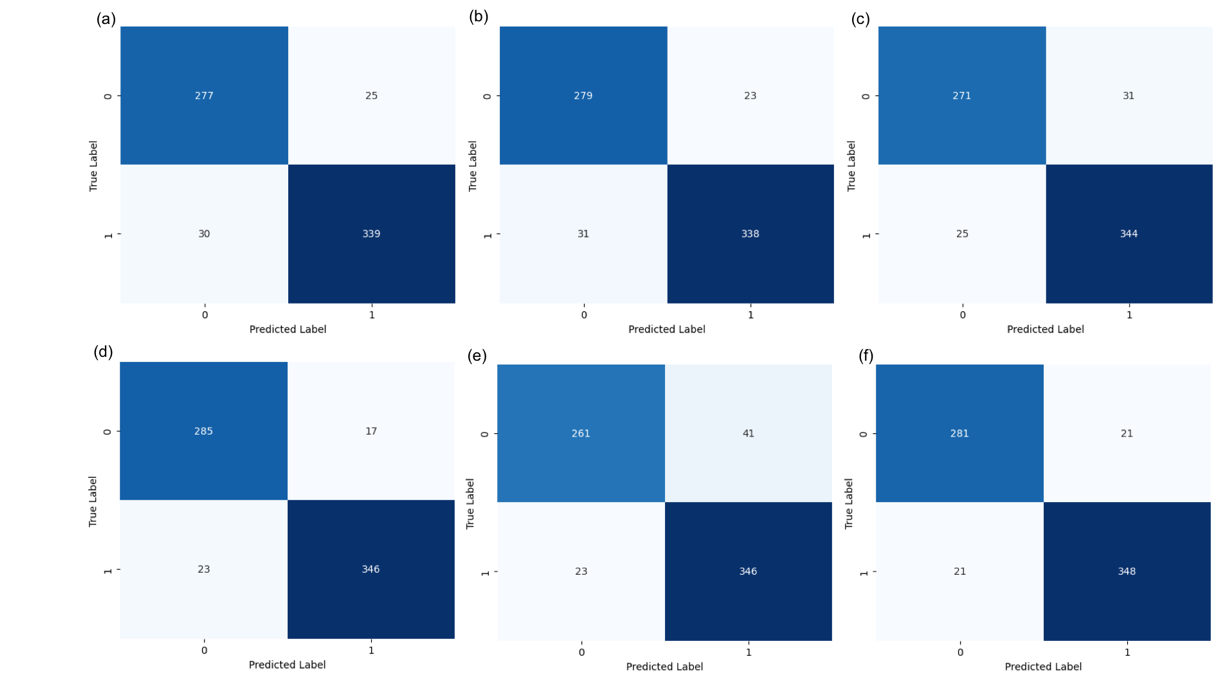


**Figure S2. Confusion matrices for Escherichia coli under leave-one-species-out (LOSO) evaluation across progressively enriched feature configurations.**

Panels correspond to the following feature sets:

(a) sequence-derived baseline features (gene length, intergenic distance, GC content difference, strand orientation);

(b) baseline + conservation;

(c) baseline + functional annotation;

(d) baseline + conservation + functional annotation;

(e) baseline + conservation + functional annotation + protein family;

(f) baseline + conservation + functional annotation + protein family + STRING interaction score.

Rows indicate true labels (0 = non-operonic, 1 = operonic), and columns indicate predicted labels.


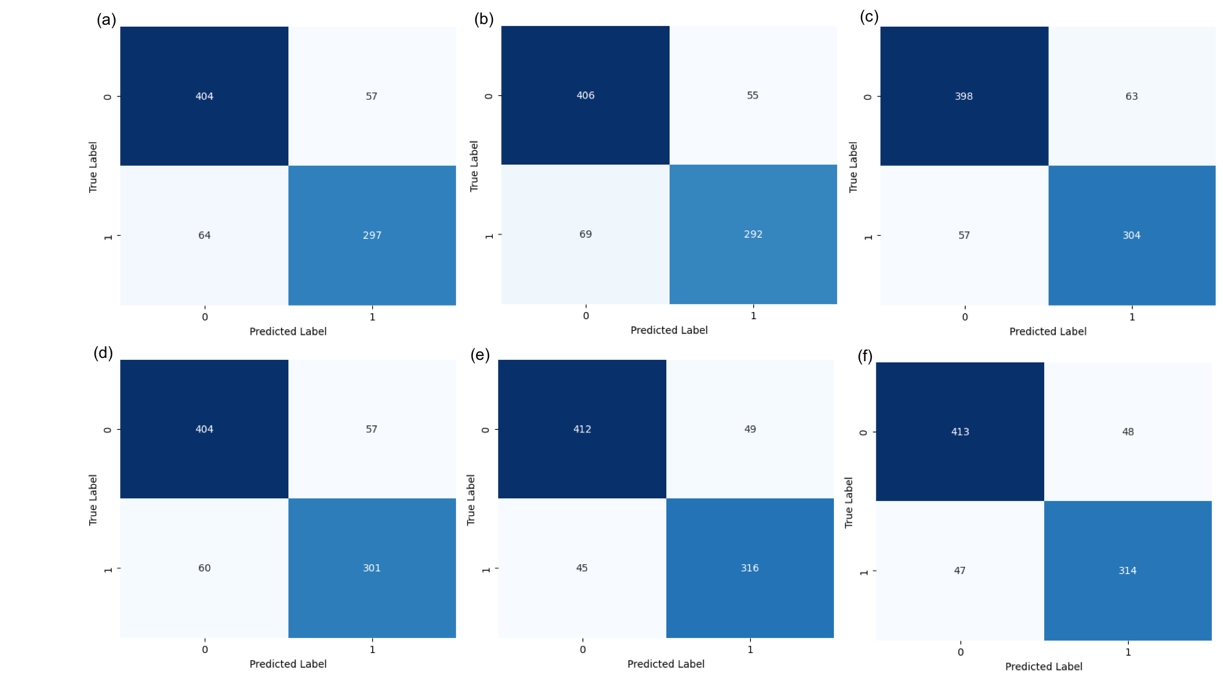

Supplement: vbag140_Supplementary_Data [file vbag140_supplementary_data.docx]
